# Gestational Weight Gain in Pregnancies Following Bariatric Surgery

**DOI:** 10.1007/s11695-023-06496-4

**Published:** 2023-02-22

**Authors:** Christos Iacovou, Tanya Maric, Miriam Bourke, Deesha Patel, Makrina Savvidou

**Affiliations:** 1 Academic Department of Obstetrics and Gynaecology, Department of Metabolism, Digestion and Reproduction, Imperial College London, Chelsea & Westminster Hospital, 369 Fulham Road, SW10 9NH London, UK; 2 Fetal Medicine Unit, Chelsea and Westminster Hospital, 369 Fulham Road, London, SW10 9NH UK

**Keywords:** Bariatric surgery, Obesity, Gestational weight gain, Pregnancy

## Abstract

**Introduction:**

To compare the gestational weight gain (GWG) between women with previous bariatric surgery and those without and investigate whether GWG correlates with birthweight (BW) or delivery of a small-for-gestational-age (SGA) neonate.

**Materials and Methods:**

Prospective, longitudinal study, include 100 pregnant women with previous bariatric surgery and 100 without weight loss surgery, but with similar early-pregnancy body mass index (BMI). In a sub-study, 50 of the post-bariatric women were also matched to 50 women without surgery, but early-pregnancy BMI similar to the pre-surgery BMI of the post-bariatric ones. All women had their weight/BMI measured at 11–14 and 35–37 weeks of gestation, and the difference in maternal weight/BMI between the two time points was expressed as GWG/BMI gain. Associations between maternal GWG/BMI gain and birthweight (BW) were examined.

**Results:**

Compared to no bariatric women with similar early-pregnancy BMI, post-bariatric women had similar GWG (*p* = 0.46), and the number of women with appropriate, insufficient, and excessive weight gain was comparable between groups (*p* = 0.76). However, post-bariatric women delivered smaller babies (*p* < 0.001), and GWG was not a significant predictor of BW or of delivering a SGA neonate. Compared to no bariatric women with similar pre-surgery BMI, post-bariatric ones had higher GWG (*p* < 0.01) but still delivered smaller neonates (*p* = 0.001).

**Conclusions:**

Post-bariatric women seem to have similar or greater GWG compared to women without surgery matched for early-pregnancy or pre-surgery BMI, respectively. Maternal GWG was not associated with BW or higher prevalence of SGA neonates seen in women with previous bariatric surgery.

**Graphical Abstract:**

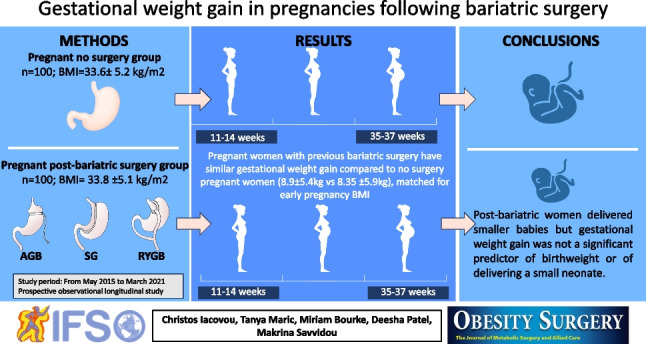

## Introduction

The global pandemic of obesity is on the rise and has nearly tripled from 1975–2016 with 39% of the global adult population being overweight [1]. In 2019, a health survey for England deemed 31% of women overweight and 29% obese [2], whereas in the US 1 in 2 women of childbearing age are classified as either overweight or obese [3]. Obesity in pregnancy is associated with an increased risk of adverse outcomes such as pre-eclampsia (PE), gestational diabetes mellitus (GDM), large-for-gestational-age (LGA) neonates, emergency Caesarean section, and stillbirth [4–6]. In contrast, pregnant women with low body mass index (BMI) are at increased risk of preterm birth and delivery of small-for-gestational-age (SGA) neonates [7]. Apart from the importance of booking BMI, emphasis has also been given on the role of gestational weight gain (GWG) in pregnancy outcomes [8, 9]. The general consensus on the optimal GWG is based on the Institute of Medicine (IOM) guidelines which state that pregnant women with normal BMI (18.5–24.9 kg/m^2^), overweight (BMI = 25–29.9 kg/m^2^), and obese (BMI ≥ 30 kg/m^2^) should gain 11.5–16 kg, 7–11.5 kg, and 5–9 kg, respectively [8]. Women who gain weight according to IOM guidelines are more likely to have better maternal and perinatal outcomes [10], including reduced rates of LGA and SGA neonates and reduced incidence of maternal hypertension, Caesarean section, and neonatal hypoglycaemia [11]. Inadequate GWG has been shown to increase the risk of having a SGA neonate, particularly in underweight women, whereas excessive GWG, combined with a raised maternal BMI, has been shown to increase the risk of a LGA neonate and low Apgar scores but decrease the risk of delivering a SGA neonate [12].

The dramatic increase in rates of obesity has led to an increase in demand for bariatric surgery, which is the most successful treatment for long-lasting weight loss and obesity related co-morbidities [13]. Worldwide, approximately 70–80% of patients undergoing bariatric surgery are women [14, 15] with up to 40% of them being of childbearing age [15, 16], meaning that an increasing number of these patients are likely to require antenatal care. Studies have suggested that pregnancy following bariatric surgery is associated with lower rates of LGA neonates but increased rates of SGA neonates and preterm delivery, compared to pregnancies in women with no surgery but booking BMI similar to the early-pregnancy or pre-surgery BMI of the post-bariatric ones [17]. A relatively small number of studies have investigated the effects of maternal GWG on the outcome of post-bariatric pregnancies, and especially on birthweight (BW) and increased rates of SGA neonates, seen in this population [18–24]. However, most of these studies were retrospective and therefore unable to evaluate reliably the role of GWG in this population.

The aim of the current study was to investigate, prospectively, the impact of GWG on pregnancy outcomes in women with previous bariatric surgery and also compare it to those of women without history of weight loss surgery.

## Material and Methods

This study was part of a larger prospective, longitudinal study investigating the impact of bariatric surgery on maternal and perinatal outcomes. The study protocol has been previously published [25, 26]. In brief, women with previous bariatric surgery, and those without, were recruited in the first trimester of pregnancy and seen at five time points during pregnancy (11–14, 20–24, 28–30, 30–32, 35–37 weeks) and at delivery. At recruitment, maternal characteristics such as age, parity, ethnicity, method of conception, smoking status, previous medical/obstetric history, and details of bariatric surgery (where applicable) were recorded in our research database. All pregnancies were singletons and dated by crown-rump length. At each encounter, maternal weight, to the nearest 0.1 kg using a calibrated electronic scale (Marsden Scales), and height, to the nearest 0.5 cm, were measured with the women in light clothing and without shoes. These measurements were used to calculate the maternal BMI (kg/m^2^) at each visit. All women underwent screening for GDM at 28–30 weeks of gestation; all no surgery patients underwent a 75 g, 2-h oral glucose tolerance test (OGTT), and GDM was diagnosed according to NICE guidelines [27]. All post-bariatric women had fasting blood glucose levels measured and 2 weeks of home glucose monitoring. Hypertensive disorders in pregnancy (BP ≥ 140/90 + / − proteinuria) were diagnosed and managed as per NICE guidelines [28]. All women delivered a live, phenotypically normal neonate. Data on pregnancy outcome were obtained from the hospital records. The neonatal BW was measured to the nearest gram, soon after delivery, using an electronic weighing scale (Seca Model 384, Germany), and BW percentiles were calculated based on gestational age at delivery and BW [29]. Small-for-gestational-age and LGA neonates were defined as delivery of a neonate with BW < 10th and > 90th percentile, respectively.

Post-bariatric pregnant women were matched with women, with no history of bariatric surgery, but with similar early-pregnancy BMI. In a sub-study, some of the post-bariatric women were matched to no surgery women with booking BMI similar to their pre-surgery BMI. Women were also matched for age, ethnic group, and parity. We only included women who had their weight/BMI measured in the first (11–14 weeks) and third (35–37 weeks) trimester of pregnancy. Gestational weight gain was calculated by subtracting the weight at 11–14 weeks from the weight at 35–37 weeks giving the total GWG. The total GWG was also categorized into appropriate, insufficient, or excessive, as per IOM classification [13]. Using the above calculations, the total BMI gain was also calculated. For post-bariatric women only, we calculated the surgery to conception interval, as the time (in months) between surgery and conception which was defined as the 14th day of pregnancy, and weight loss, expressed as BMI (kg/m^2^) loss from pre-surgery (self-reported) to the first trimester of pregnancy. The study was approved by the West London Research Ethics Committee (No: 14/LO0592-2014 on 03/06/2014), and all women gave written informed consent.

### Statistical Analysis

Normality of the distribution of the data was examined with the Kolmogorov–Smirnov test. Logarithmic transformation was performed for no normally distributed data. Data were expressed as mean (standard deviation (SD)) or as median (interquartile range) for normally and no normally distributed data, respectively. Comparisons between groups were performed using unpaired Student’s *t*-test/Mann–Whitney *U*-test or chi-square test for numerical and categorical data, respectively. Pearson’s correlation was used to examine the relation between maternal characteristics and BW. Using the significant predictors, a multiple regression was then created to evaluate which factors remained significant predictors of BW. Binomial logistic regression was used to derive the association between maternal characteristics, including GWG, and delivery of a SGA neonate. Statistical analyses were performed using the Statistical Package for the Social Sciences (SPSS for Windows 2019, version 26.0, IBM Corp., Armonk, New York, US), and differences were considered significant at *p* < 0.05.

## Results

  The study period was from May 2015 to March 2021 and included 100 post-bariatric pregnant women who were matched to 100 pregnant women with no weight loss surgery history but with similar early-pregnancy BMI. The post-bariatric group included 21 women who had undergone a gastric band procedure, 30 with sleeve gastrectomy, and 49 with gastric bypass. The mean surgery to conception interval was approximately 4.5 years (55.89 (36.13) months) and only 8 women conceived within 12 months of the surgery. The mean BMI loss was 12.18 (6.32) kg/m^2^. The demographic characteristics, biophysical profile, and pregnancy outcomes of the study participants, according to the type of surgery performed, are given in Table 1.

**Table 1:** Demographic characteristics, biophysical measurements and pregnancy outcomes of the post-bariatric and no surgery women matched for early-pregnancy maternal body mass index. All comparisons were made with the no surgery group

Outcomes	No surgery (*n* = 100)	Post-bariatric surgery (*n* = 100)	*p* value	Post-gastric band (*n* = 21)	Post-sleeve gastrectomy (*n* = 30)	Post-gastric bypass (*n* = 49)
Maternal age (years)	32.54 (5.02)	33.85 (5.02)	0.07	33.10 (4.15)	33.90 (5.07)	34.14 (5.39)
Parity, *n* (%)			0.88			
Nulliparous	50 (50.0)	49 (49.0)		12 (57.1)	17 (56.7)	20 (40.8)
Parous	50 (50.0)	51 (51.0)		9 (42.9)	13 (43.3)	29 (59.2)
Ethnic group, *n* (%)			0.76			
White	65 (65.0)	67 (67.0)		10 (47.6)	23 (76.7)	34 (69.4)
Other	35 (35.0)	33 (33.0)		11 (52.4)	7 (23.3)	15 (30.6)
Conception, *n* (%)			0.42			
Spontaneous	91 (91.0)	94 (94)		20 (95.2)	30 (100.0)	44 (89.8)
Assisted reproductive techniques	9 (9.0)	6 (6.0)		1 (4.8)	0 (0)	5 (10.2)
Smoking, *n* (%)			0.18			
No	95 (95.0)	90 (90.0)		19 (90.5)	27 (90.0)	44 (89.8)
Yes	5 (5.0)	10 (10.0)		2 (9.5)	3 (10.0)	5 (10.2)
BMI prior to surgery (kg/m^2^)	-	45.82 (7.23)	-	44.10 (8.99)	43.51 (6.72)	47.97 (6.12)
Booking weight (kg)	90.74 (15.81)	92.81 (16.80)	0.37	102.84 (19.25)*	89.23 (14.63)	90.69 (15.55)
Booking BMI (kg/m^2^)	33.61 (5.17)	33.76 (5.07)	0.83	36.76 (5.39)*	32.76 (4.68)	33.08 (4.77)
Weight at 36 weeks (kg)	99.09 (15.52)	101.75 (16.63)	0.24	112.40 (20.52)*	98.36 (14.68)	99.26 (14.19)
BMI at 36 weeks (kg/m^2^)	36.66 (4.90)	37.06 (5.21)	0.58	40.27 (6.49)*	36.13 (4.95)	36.25 (4.25)
Gestational weight gain (12–36 wks) (kg)	8.35 (5.85)	8.93 (5.38)	0.46	9.55 (7.34)	9.10 (5.34)	8.57 (4.44)
Gestational BMI gain (12–36 wks) (kg/m^2^)	3.05 (2.16)	3.30 (2.04)	0.40	3.50 (2.89)	3.37 (2.0)	3.16 (1.61)
IOM—weight gain			0.76			
Appropriate, *n* (%)	27 (27)	31 (31)		5 (23.8)	11 (36.7)	15 (30.6)
Insufficient, *n* (%)	32 (32)	28 (28)		7 (33.3)	7 (23.3)	14 (28.6)
Excessive, *n* (%)	41 (41)	41 (41)		9 (42.9)	12 (40.0)	20 (40.8)
Gestational age at delivery (wks)	39.21 (1.32)	38.98 (1.35)	0.22	39.12 (1.53)	39.15 (1.48)	38.82 (1.18)
Birthweight (g)	3444.03 (486.79)	3174.90 (488.97)	< 0.01	3298.47 (465.34)	3223.10 (483.37)*	3092.43 (496.66)*
Birthweight percentile	57.45 (31.07)	40.59 (30.52)	< 0.01	46.12 (29.96)	43.12 (32.12)*	36.67 (29.86)*
SGA neonates, *n* (%)	14 (14)	21 (21)	0.19	2 (9.5)	7 (23.3)	12 (24.5)
LGA neonates, *n* (%)	15 (15)	7 (7)	0.07	3 (14.3)	2 (6.7)	2 (4.1)*
Mode of delivery, *n* (%)			0.32			
Vaginal delivery	48 (48%)	55 (55)		12 (57.1)	15 (50.0)	28 (57.1)
Caesarean section	52 (52%)	45 (45)		9 (42.9)	15 (50.0)	21 (42.9)
Hypertensive disorders, *n* (%)			0.32			
No	89 (89.0)	93 (93.0)		18(85.7)	28 (93.3)	47 (95.9)
Yes	11 (11.0)	7 (7)		3 (14.3)	2 (6.7)	2 (4.1)
Diabetes mellitus, *n* (%)			0.43			
No	83 (83.0)	77 (77.0)		14 (66.7)	22 (78.3)	41 (83.7)
Yes	17 (17.0)	20 (23.0)		7 (33.3)	8 (26.7)	8 (16.3)

Data are expressed as mean (standard deviation), median (interquartile range), or number (%) and *p* value column for comparison between the no surgery and post-bariatric groups

*BMI* body mass index, *IOM* Institute of Medicine, *Large-for-gestational-age neonates* neonates born with birthweight > 90th percentile, *Small-for-gestational-age neonates* neonates born with birthweight < 10th percentile

^*^Denotes *p* < 0.05

The total GWG and BMI gain (12 to 36 weeks) was comparable between the groups (Table 1). Likewise, the number of women who had appropriate, insufficient, and excessive GWG, according to IOM, was similar between the groups and among women with different types of bariatric surgery. In the post-bariatric group, there was no significant difference in surgery to conception interval between women with appropriate, insufficient, or excessive GWG (*p* = 0.96). Despite similar GWG, post-bariatric women (especially those that had undergone a sleeve gastrectomy or a gastric bypass) delivered smaller babies and tended to have more SGA and less LGA babies, although these differences did not reach a significance in our population. The Pearson correlation revealed that maternal age (*p* < 0.01), ethnic group (*p* = 0.07), total GWG (*p* < 0.05), previous bariatric surgery (*p* < 0.01), and gestational age at delivery (*p* < 0.01) were significant determinants of BW, whereas maternal smoking (*p* = 0.54), method of conception (*p* = 0.46), parity (*p* = 0.35), presence of diabetes (*p* = 0.45), or hypertensive disorders (*p* = 0.41) were not. Using only the significant predictors, a multiple regression was then constructed, and maternal ethnic group (*p* = 0.09), previous bariatric surgery (*p* < 0.01), and gestational age at delivery (*p* < 0.01) remained significant predictors of BW, whereas maternal age and GWG were not. The results were unchanged when, instead of GWG (in kg), maternal BMI gain or GWG (according to IOM category) were considered. Furthermore, insufficient or excessive GWG, according to IOM guidelines, was not an independent predictor of delivering a neonate that was SGA or LGA, respectively. We then considered the groups separately and, in the no surgery group, we found that although GWG, as a continuous variable, was not associated with BW (*p* = 0.27), insufficient GWG was a significant independent predictor of delivering a SGA neonate (*p* = 0.02) (Table 3). In the post-bariatric group as a whole, we also found no association between maternal GWG and BW (*p* = 0.52), and this was the case even if when different types of surgery were considered separately (*p* = 0.67, *p* = 0.93, and *p* = 0.61 for women with a gastric band, sleeve gastrectomy, and gastric bypass, respectively). We also found no correlation between insufficient GWG and delivery of a SGA neonate in the post-bariatric group, as a whole (Table 3), or when women with a gastric band (*p* = 0.29), sleeve gastrectomy (*p* = 0.71), and gastric bypass (*p* = 0.75) were considered separately. Additionally, there was no independent association (in the whole cohort or in the groups separately) between GWG and gestational age at delivery (*p* = 0.12), when maternal characteristics such as age, parity, method of conception, and presence of diabetes were taken into account.

In the sub-study, 50 post-bariatric (12 with gastric band, 13 with gastric sleeve, and 25 with gastric bypass) pregnant women were matched to 50 women with no surgery but with early-pregnancy BMI similar to the pre-surgery BMI of the post-bariatric ones (43.90 (7.35) kg/m^2^ vs 43.19 (7.53) kg/m^2^; *p* = 0.63). For post-bariatric women, the mean surgery to conception interval was almost 4.5 years (53.42 (31.40) months), and the mean BMI loss was 11.69 (7.19) kg/m^2^. The demographic characteristics, biophysical profile, and pregnancy outcomes of the study participants are given in Table 2. Post-bariatric women were more likely to be smokers and had lower weight/BMI at both time points (first and third trimesters), as expected, but gained more weight/BMI during pregnancy. Although, post-bariatric women gained more weight, they still delivered smaller babies and tended to have higher rates of SGA and lower rates of LGA neonates. Pearson correlation showed that in the whole sub-study cohort, maternal age (*p* < 0.01), previous bariatric surgery (*p* = 0.01), and gestational age at delivery (*p* < 0.01) were significant predictors of BW, but maternal ethnic group (*p* = 0.9), smoking (*p* = 0.16), method of conception (*p* = 0.11), parity (*p* = 0.82), total GWG (*p* = 0.78), presence of diabetes (*p* = 0.36), and hypertensive disorders (*p* = 0.19) were not. Using only the significant predictors, a multiple regression showed that previous maternal bariatric surgery (*p* < 0.01) and gestational age at delivery (*p* < 0.01) remained significant independent predictors of BW. In the whole sub-study cohort, insufficient or excessive GWG was not an independent predictor of delivering a SGA or LGA neonate, respectively. We did not consider the no surgery and post-bariatric groups separately, as due to the small sample size, we could not compute the association of GWG categories with the risk of delivering a SGA or LGA neonate; none of the SGA infants was born in post-bariatric women with insufficient GWG (Table 3). Moreover, there was no independent association between GWG and gestational age at delivery (*p* = 0.80) when maternal characteristics were considered.

**Table 2:** Demographic characteristics, biophysical measurements, and pregnancy outcomes of the no surgery and post-bariatric women matched for pre-surgery body mass index of the latter group. All comparisons were made with the no surgery group

Outcomes	No surgery (*n* = 50)	Post-bariatric surgery (*n* = 50)	*p* value	Post-gastric band (*n* = 12)	Post-sleeve gastrectomy (*n* = 13)	Post-gastric bypass (*n* = 25)
Maternal age (years)	32.54 (4.70)	33.48 (4.48)	0.31	34.0 (4.78)	33.46 (3.75)	33.24 (4.83)
Parity, *n* (%)			0.42			
Nulliparous	20 (40.0)	24 (48.0)		7 (58.3)	7 (53.8)	10 (40.0)
Parous	30 (60.0)	26 (52.0)		5 (41.7)	6 (46.2)	15 (60.0)
Ethnic group, *n* (%)			0.51			
White	36 (72.0)	33 (66.0)		5 (41.7)*	10 (76.9)	18 (72.0)
Other	14 (28.0)	17 (34)		7 (58.3)	3 (23.1)	7 (28.0)
Conception, *n* (%)			0.40			
Spontaneous	48 (96.0)	46 (92.0)		11 (91.7)	13 (100.0)	22 (88.0)
Assisted reproductive techniques	2 (4.0)	4 (8.0)		1 (8.3)	0 (0)	3 (12.0)
Smoking, *n* (%)			0.05			
No	49 (98.0)	44 (88.0)		11 (91.7)	12 (92.3)	21 (84.0)*
Yes	1 (2.0)	6 (12.0)		1 (8.3)	1 (7.7)	4 (16.0)
Booking weight (kg)	114.53 (21.75)	88.00 (16.84)	< 0.01	98.70 (22.13)*	81.06 (10.24)*	86.46 (14.70)*
Booking BMI (kg/m^2^)	43.19 (7.53)	32.20 (5.22)	< 0.01	35.61 (6.04)*	30.27 (4.15)*	31.57 (4.70)*
Weight at 36 weeks (kg)	120.74 (21.76)	97.28 (17.23)	< 0.01	108.34 (24.46)	90.60 (10.20)*	95.45 (13.82)*
BMI at 36 weeks (kg/m^2^)	45.50 (7.20)	35.59 (5.31)	< 0.01	39.13 (7.08)*	33.80 (3.92)*	34.82 (4.27)*
Gestational weight gain (12–36 wks) (kg)	6.21 (5.15)	9.28 (4.89)	< 0.01	9.63 (6.19)	9.53 (4.79)*	8.98 (4.40)*
Gestational BMI gain (12–36 wks)	2.32 (1.99)	3.39 (1.79)	< 0.01	3.51 (2.34)	3.53 (1.71)*	3.26 (1.58)*
IOM-Weight gain			0.55			
Appropriate, *n* (%)	18 (36.0)	20 (40.0)		3 (25.0)	6 (46.1)	11 (44.0)
Insufficient, *n* (%)	18 (36.0)	13 (26.0)		4 (33.3)	2 (15.4)	7 (28.0)
Excessive, *n* (%)	14 (28.0)	17 (34.0)		5 (41.7)	5 (38.5)	7 (28.0)
Gestational age at delivery (wks)	39.09 (1.46)	39.30 (1.22)	0.44	39.27 (1.52)	39.70 (1.45)	39.10 (0.89)
Birthweight (g)	3522.54 (525.22)	3249.14 (513.43)	0.01	3251.67 (543.57)	3325.92 (555.21)	3208.00 (493.65)*
Birthweight percentile	64.11 (29.80)	43.09 (32.43)	< 0.01	39.78 (33.14)*	46.59 (37.54)	42.85 (30.44)*
SGA neonates, *n* (%)	3 (6.0)	9 (18.0)	0.06	1 (8.3)	4 (30.8)*	4 (16.0)
LGA neonates, *n* (%)	13 (26.0)	6 (12.0)	0.07	2 (16.7)	2 (15.4)	2 (8.0)
Mode of delivery, *n* (%)						
Vaginal delivery	28 (56.0)			5 (41.7)	8 (61.5)	
Caesarean section	22 (44.0)			7 (58.3)	5 (38.5)	
Hypertensive disorders, *n* (%)			0.33			
No	43 (86.0)	46 (92.0)		9 (75.0)	13 (100.0)	24 (96.0)
Yes	7 (14.0)	4 (8.0)		3 (25.0)	0 (0)	1 (4.0)
Diabetes mellitus, *n* (%)			0.04			
No	33 (66.0)	42 (84.0)		9 (75.0)	10 (76.9)	23 (92.0)*
Yes	17 (34.0)	8 (16.0)		3 (25.0)	3 (23.1)	2 (8.0)

Data are expressed as mean (standard deviation), median (interquartile range), or *n* (%)

*BMI* body mass index, *IOM* Institute of Medicine, *LGA neonates* neonates with birthweight > 90th percentile, *SGA neonates* neonates with birthweight < 10th percentile

^*^Denotes *p* < 0.05

**Table 3:** Distribution of small-for-gestational-age and large-for-gestational-age neonates in the study groups (4) according to the maternal gestational weight gain categories based on the Institute of Medicine guidelines

	No surgery group (*n* = 100)		Post-bariatric group (*n* = 100)		No surgery group (*n* = 50)		Post-bariatric group (*n* = 50)	
	Matched for early-pregnancy body mass index				Matched for pre-surgery body mass index			
	SGA	LGA	SGA	LGA	SGA	LGA	SGA	LGA
Total (*n*%)	14 (14%)	15 (15%)	21 (21%)	7 (7%)	3 (6%)	13 (26%)	9 (18%)	6 (12%)
Insufficient GWG, *n*	7	4	5	3	1	3	0	2
Adequate GWG, *n*	5	4	9	2	2	7	6	2
Excessive GWG, *n*	2	7	7	2	0	3	3	2

*GWG* gestational weight gain, *LGA* large-for-gestational-age neonates with birthweight > 90th percentile, *SGA* small-for-gestational-age neonates with birthweight < 10th percentile

## Discussion

This prospective observational study has demonstrated that the trimester-specific GWG of post-bariatric and no surgery women, matched for early-pregnancy weight/BMI, is similar. Nevertheless, the former group delivered smaller neonates. Furthermore, the distribution of women who gained appropriate, insufficient, or excessive weight during pregnancy, according to IOM guidelines, was comparable in both groups. Maternal GWG was not associated with BW, and although insufficient GWG was a significant predictor of delivering a SGA in the no surgery group, this was not the case in the post-bariatric group. When post-bariatric pregnant women were compared to women with early-pregnancy weight/BMI similar to their pre-surgery weight/BMI, we found that the former group had greater GWG but still delivered smaller neonates.

Gestational weight gain has generally been shown to have an impact on pregnancy outcomes. A recent meta-analysis including more than one million pregnant women has demonstrated that insufficient GWG was associated with higher risk of having a SGA neonate and preterm birth, whereas excessive GWG was associated with increased risk of LGA neonates, macrosomia, Caesarean delivery, and reduced risk of SGA and preterm delivery [9]. In consistence with that report, we found that insufficient GWG was associated with increased risk of delivering a SGA neonate but only in the no surgery group. In contrast, maternal insufficient GWG was not associated with the delivery of a SGA neonate in the post-bariatric group. Most of the studies on GWG in post-bariatric pregnant women are retrospective; reported maternal weight at different gestational weeks/times or “the last known weight of pregnancy”; used different control groups, if any; and reported conflicting results [18–24]. One retrospective study examining the effect of trimester-specific GWG on BW, in women with previous biliopancreatic diversion, found that although women had less GWG, compared to women without surgery, there was still no association between GWG and BW [19]. Other retrospective studies, including women with laparoscopic adjustable gastric banding, sleeve gastrectomy, or gastric bypass, showed similar GWG between post-bariatric and no surgery women [20, 21] when a control group was used, but then again, there was lack of an association between GWG and risk of delivering a SGA neonate [20, 22]. In contrast, other retrospective studies have shown an association between GWG and BW [18, 24]. The only prospective study in women with previous bariatric surgery has reported an association between insufficient GWG, defined as the difference between “measured weight at the time of childbirth” and self-reported pre-conception weight, and delivery of a SGA neonate, as well as preterm birth [23]. However, the study did not include a no surgery comparative group, and the lack of standardization of the gestation at which the maternal weight was measured may have introduced bias, as women who delivered earlier had, certainly, less GWG compared to the ones who delivered later. Conversely, in our study we calculated the GWG using a standardized approach of measuring the maternal weight at 12 and 36 weeks, for all patients.

In our study, women with previous bariatric surgery delivered significantly smaller babies and had more SGA and less LGA neonates, compared to women with no surgery, even if these differences did not always reach statistical significance. Maternal GWG does not seem to play a role in determining BW in the post-bariatric group, and therefore, it is probable that, in this group, other mechanisms are involved, including specific maternal nutritional deficiencies and/or alteration of the maternal metabolic/glucose environment [25, 26, 30]. However, nutritional levels of post-bariatric women with small and normal size neonates are reported to be similar [31]. Furthermore, if GWG is used as a surrogate for the general maternal nutritional status and considering that, in our study, post-bariatric women had similar or even greater GWG, than their relevant counterparts, then it is unlikely that nutrition will be proven to be a key determinant of BW. However, maternal glucose levels/variation appears more likely to play a role in determining the BW in this high-risk population. Of note, pregnant women with previous bariatric surgery have been shown to have lower fasting glucose and insulin resistance levels, compared to women without surgery, and these levels have been positively associated with BW and body fat deposition of their offspring [25, 26].

In our study, the proportion of post-bariatric women who gained insufficient weight was 28%, which is similar to the proportion reported in another prospective study [23] but lower to what has been reported in other studies [14, 19]. This may be due to the fact that most of our post-bariatric participants conceived many years after the surgery and therefore were not in the catabolic, post-operative phase, which has been associated with insufficient GWG [32], or because most of the previous studies included women from more than a decade ago, when the antenatal care may not have been as optimal as it is at present times.

Our study was a single-center, prospective, longitudinal study that used robust methodology in reporting the maternal weight at specific times in pregnancy, from the first to the third trimester, and under controlled conditions. By including the maternal weight at 36 weeks, we excluded, by definition, all women that may have delivered earlier, and therefore, we were not able to assess comprehensively the potential effect of GWG on the risk of preterm delivery. The groups were closely matched not only in terms of weight/BMI but other maternal characteristics, which allowed us to minimize the impact of known confounders. The relatively small number of post-bariatric women may have prevented us from determining the effect of GWG on BW according to the type of surgery performed. Although we suggest that maternal nutrition is unlikely to affect BW, we were unable to confirm this in our population. Of note, all post-bariatric women and those with no surgery and booking BMI ≥ 35 kg/m^2^ received dietary advice and approximately 50% of the former and 66% of the latter group were on prenatal multivitamin supplementation in the first trimester, which reduced to 27% and 35%, respectively, by the third trimester. The rest of the women took prenatal vitamins separately, including folate, vitamin D, vitamin B12, and vitamin C. Furthermore, food, calorie intake, and physical activity diaries were not recorded during our study.

In conclusion, this prospective study has shown that pregnant women with previous bariatric surgery have similar or greater GWG compared to no surgery pregnant women matched for early-pregnancy and pre-surgery BMI, respectively. However, the GWG of post-bariatric women does not seem to be associated with BW or the higher prevalence of SGA neonates seen in this population. Furthermore, studies are required to investigate the determinants of BW in this high-risk group.

## Data Availability

The data that support this study are available on request from the corresponding author.
